# The Chromosome-Level Genome Assembly of Bean Blossom Thrips (*Megalurothrips usitatus*) Reveals an Expansion of Protein Digestion-Related Genes in Adaption to High-Protein Host Plants

**DOI:** 10.3390/ijms241411268

**Published:** 2023-07-10

**Authors:** Zhijun Zhang, Jiandong Bao, Qizhang Chen, Jianyun He, Xiaowei Li, Jiahui Zhang, Zhixing Liu, Yixuan Wu, Yunsheng Wang, Yaobin Lu

**Affiliations:** 1 State Key Laboratory for Managing Biotic and Chemical Threats to the Quality and Safety of Agro-Products, Institute of Plant Protection and Microbiology, Zhejiang Academy of Agricultural Sciences, Hangzhou 310021, China; baojd@zaas.zc.cn (J.B.); 17300906030@163.com (Q.C.); hjy12242021@163.com (J.H.); lixiaowei1005@163.com (X.L.); 15096100996zjh@gmail.com (J.Z.); 13271305165@163.com (Z.L.); wyx0425123@163.com (Y.W.); 2 Hunan Provincial Key Laboratory for Biology and Control of Plant Diseases and Insect Pests, Hunan Agricultural University, Changsha 410125, China; wyunsheng@gmail.com

**Keywords:** *Megalurothrips usitatus*, bean blossom thrips, chromosome-level genome assembly, paralog, protein digestion-related genes, legumes

## Abstract

*Megalurothrips usitatus* (Bagnall) is a destructive pest of legumes, such as cowpea. The biology, population dynamics and control strategies of this pest have been well studied. However, the lack of a high-quality reference genome for *M. usitatus* has hindered the understanding of key biological questions, such as the mechanism of adaptation to feed preferentially on high-protein host plants and the resistance to proteinase inhibitors (PIs). In this study, we generated a high-resolution chromosome-level reference genome assembly (247.82 Mb, 16 chromosomes) of *M. usitatus* by combining Oxford Nanopore Technologies (ONT) and Hi-C sequencing. The genome assembly showed higher proportions of GC and repeat content compared to other Thripinae species. Genome annotation revealed 18,624 protein-coding genes, including 4613 paralogs that were preferentially located in TE-rich regions. GO and KEGG enrichment analyses of the paralogs revealed significant enrichment in digestion-related genes. Genome-wide identification uncovered 506 putative digestion-related enzymes; of those, proteases, especially their subgroup serine proteases (SPs), are significantly enriched in paralogs. We hypothesized that the diversity and expansion of the digestion-related genes, especially SPs, could be driven by mobile elements (TEs), which promote the adaptive evolution of *M. usitatus* to high-protein host plants with high serine protease inhibitors (SPIs). The current study provides a valuable genomic resource for understanding the genetic variation among different pest species adapting to different plant hosts.

## 1. Introduction

*Megalurothrips usitatus* (Bagnall, commonly known as bean flower thrips) is a small-sized (~2 mm) destructive pest of vegetable crops, particularly legumes such as cowpea, in Asia [1]. The entire life cycle of the host plant, especially the flowering stage, is affected by *M. usitatus* feeding on flowers, leaves and fruits, resulting in the necrosis and premature abortion of buds and flowers [2]. *M. usitatus* causes severe reductions in the yield, quality and economic value of crop production. Although ecologically sound and sustainable control measures are being developed to manage *M. usitatus*, control of the pest is based solely on chemical insecticides in the field [3,4]. The intensive use of chemical insecticides such as emamectin benzoate, spinetoram and beta-cypermethrin against *M. usitatus* has resulted in high levels of insecticide resistance in this pest [5,6]. The damage caused by *M. usitatus* has increased over the years and has become a major pest of cowpea (*Vigna unguiculata* ssp. *sesquipedalis*) in southern China and, more recently, an invasive pest of snap bean (*Phaseolus vulgaris* L.) in the Americas [7].

*M. usitatus* belongs to Thripidae, a family of Thysanoptera (also known as thrips) that comprises over 7000 species, many of which are pests of agricultural and horticultural crops worldwide [8,9], such as *Frankliniella occidentalis*, *F. instona*, *Thrips palmi*, *T. tabaci* and *M. usitatus*. To date, only a few genome assemblies of Thripidae species are available. The first species is *F. occidentalis*, which was assembled to a contig-level genome using Illumina short reads [10], while *T. palmi* is the first and only species with a chromosome-level genome assembled using PacBio long reads combined with a high-throughput chromosome conformation capture (Hi-C) strategy [11]. Although there have been some genetic analyses of *M. usitatus*, such as transcriptome and mitochondrial genome analyses [1,12], a high-quality genome assembly of this species is needed to facilitate future studies on the genetics, diversity, adaptation and evolution of thrips.

Recent advances in insect genomics have contributed greatly to our understanding of the mechanisms involved in insect host plant adaptations [13,14,15,16,17]. Genome sequencing of the green peach aphid (*Myzus persicae*) revealed an expansion of genes encoding cathepsin B-like proteins (belonging to the cysteine proteinase family) [18], which can not only digest host plant proteins to amino acids [19], but can also degrade protease inhibitors (PIs) produced by the host plants [20]. An expansion of the P450 gene family in *M. persicae* (115 genes) reflects the large host range of *M. persicae* relative to the legume-specialist aphid (*Acyrthosiphon pisum*, 83 genes) [21]. The sweet potato whitefly (*Bemisia tabaci*) shows exceptional host adaptability (more than 600 host plant species) as it harbors a plant-derived horizontally transferred gene *BtPMaT1*, which encodes a phenolic glucoside malonyltransferase that enables *B. tabaci* to detoxify phenolic glycosides present in host plants [17].

Insect digestive enzymes determine the specificity of insects’ adaptation to different host plants as food and are essential for them to survive and thrive. The mono-host plant of *M. usitatus*, cowpea (*V. unguiculata*), as with other legumes, is well known for having a high protein content [22] and also abundant proteinase inhibitors (PIs) [23] that have strong negative effects on insects through either constitutive or induced expression [24]. PIs lead to the development of three major adaptive strategies in insect pests: (1) overexpression of PI-sensitive proteinases, (2) evolution of PI-insensitive proteinases and the proteolytic cleavage of PIs to disarm them, and (3) production of PI-hydrolyzing proteinases [25]. However, to date, there is insufficient genetic information on the digestive physiology of thrips. The underlying mechanisms of *M. usitatus* that allow the digestion of high-value host proteins and defense against the abundant PIs of the host plant need to be elucidated.

In the present study, we employed long-read third-generation sequencing (TGS) and short-read next-generation sequencing (NGS) as well as Hi-C scaffolding technologies, generated a high-quality chromosome-level genome assembly and RNA-seq based on well-annotated gene resources of *M. usitatus*, explored the potential mechanism of adaptation to the host plant Leguminosae with a high quantity of proteins and PIs, and revealed the evolutionary relationship by comparative genomics with representative insects and other thrips with available genomes. The results showed that the expansion of paralogs, which significantly enriched protein digestion-related genes, driven by mobile elements may play an important role in the evolution of *M. usitatus*. The genome assembly of *M. usitatus* in this study provides a valuable genetic resource for understanding variations among different species of thrips and their evolutionary mechanisms for adaptation to different host plants.

## 2. Results

### 2.1. Genome Sequencing and Assembling of M. usitatus

**Figure 1:**
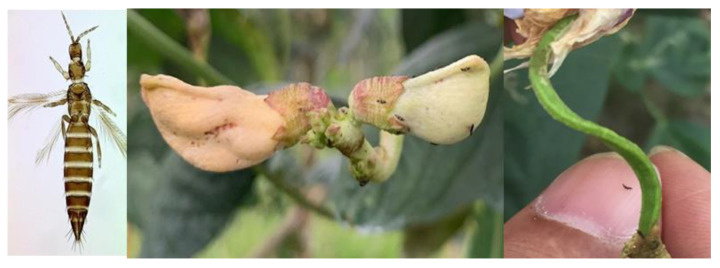
Adults of *M. usitatus* (**left**) and damage on flower (**middle**) and fruits (**right**) of *V. unguiculata*.

We deployed a combination of short-read NGS and long-read Oxford Nanopore Technologies (ONT) to sequence the genome of *M. usitatus* using pooled male and female adult samples of the bean blossom thrips *M. usitatus* (Figure 1). In total, we obtained 32.98 Gb ONT reads (coverage: 133×) with an average length of 16.40 kb for de novo genome assembly and 17.83 Gb NGS reads (coverage: 72×) for genome correction (Table S1). We obtained a 262.63 Mb genome assembly (GC content: 55.4%) for *M. usitatus* consisting of 187 contigs with N50 of 11.32 Mb (Table 1). The GC content is comparable to the published genomes of other Thripinae species (*F. occidentalis*, *T. palmi*) [10,11] and significantly higher than other insect species [26,27]. Over 15% of the genome is covered by repeat sequences, which is higher than the available genomes of the other two Thripinae species.

**Figure 2:**
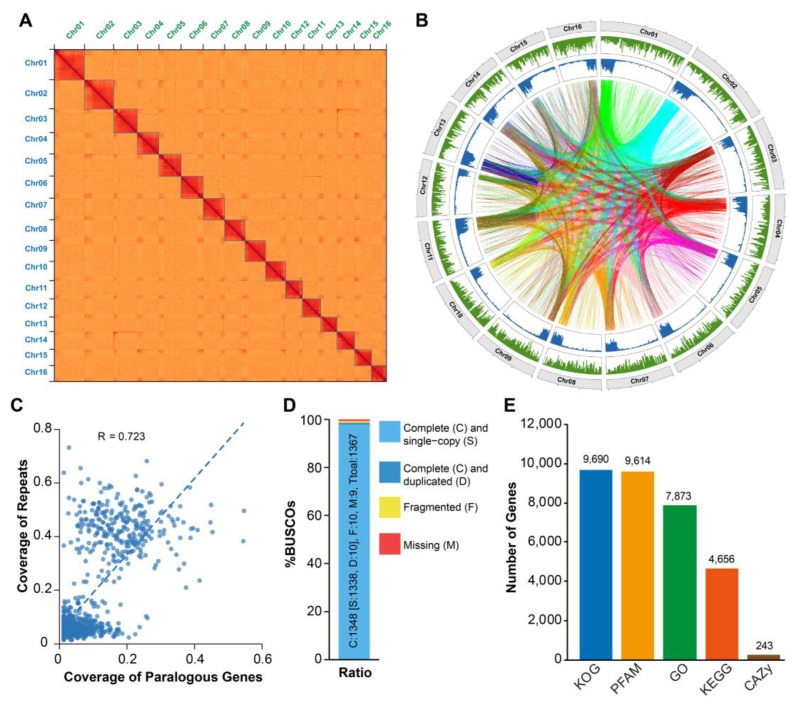
Features of chromosome-level genome assembly of *M. usitatus*: (**A**) HiC heatmap revealed 16 distinct chromosomes. (**B**) The circos display the information of *M. usitatus* genome assembly. Tracks from outside to inside represent the chromosomes and the density of repeat elements, and the linking lines connect the paralogs. (**C**) Distributions of paralogs and repeat elements show high correlation. R value represents Pearson Correlation Coefficient. (**D**) BUSCO genome completeness assessment using insecta_odb10 dataset (n = 1367). (**E**) General functionally annotated genes.

To obtain a chromosome-level genome assembly, we used 54.37 Gb (~219×) Hi-C data for scaffolding the contigs. Finally, 16 pseudochromosomes with an additional unknown scaffold were generated with a total size of 247.82 Mb, covering approximately 94.36% of the contig genome assembly (Figure 2A,B). The LTR assembly index (LAI) was used to assess assembly continuity using LTR retrotransposons, and revealed an estimated LAI value of 11.61 (Table 1), which is comparable to the LAIs of the published reference genomes such as *Arabidopsis* (TAIR10) [28]. According to the BUSCOs analysis, 1338 out of 1367 (97.88%) single-copy genes from the insecta_odb10 dataset were completely annotated (Figure 2D). In addition, NGS genomic reads were realigned to the unmasked genome assembly with a mapping rate of 96.98% (properly paired: 95.35%). These results strongly indicate the high quality, continuity and completeness of the *M. usitatus* reference genome we constructed.

### 2.2. Genome Annotation

To fully annotate the gene structure of the *M. usitatus* reference genome, we combined the results from de novo, RNA-Seq-based, and homologous-based methods. In total, we obtained 18,624 protein-coding genes with a ~3 kb average gene length and ~5 average exons. We also identified 3673 tRNAs (Table 1). We then applied five databases, including GO, KEGG, KOG, Pfam and CAZy, to functionally annotate the protein-coding genes, and this resulted in 9690, 9614, 7873, 4656 and 243 annotated genes, respectively (Table S3). In total, 10,092 protein-coding genes, representing about 54% of the total genes, were annotated with at least one functional result (Figure 2E). Nearly half of the genes could not be functionally annotated, suggesting the specificity of the gene bank of *M. usitatus*.

**Figure 3:**
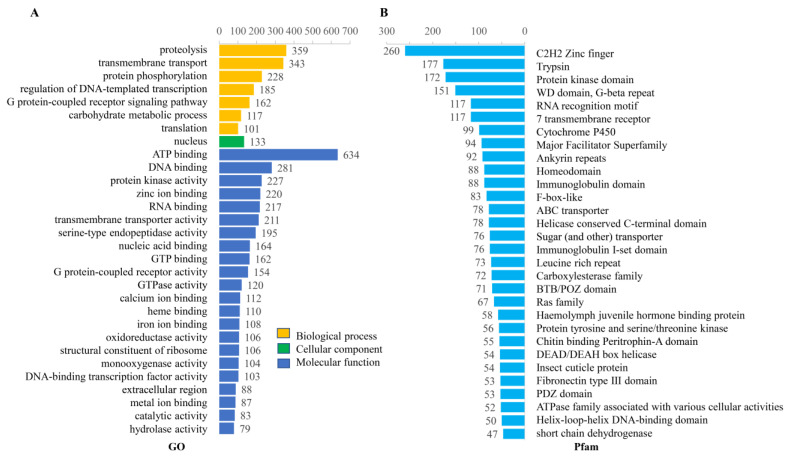
Summary of gene functional annotation of *M. usitatus* genome including GO (**A**) and Pfam (**B**). Top 30 terms are shown. Number of genes belong to the terms were shown at the top of bar.

The GO annotation revealed a number of putative digestion-related genes in the top 30 terms, such as ‘proteolysis’ (GO:0006508, 359 genes) and ‘carbohydrate metabolic process’ (GO:0005975, 117 genes) in biological processes, ‘serine-type endopeptidase activity’ (GO:0004252, 195 genes) and ‘hydrolase activity’ (GO:0016787, 79 genes) (Figure 3A and Table S4).

The Pfam annotation found that proteins with a Zinc finger domain (PF00096, 260 genes) had the highest gene number, then followed by ‘trypsin’ (PF00089, 177 genes), ‘protein kinase domain’ (PF00069, 172 genes), ‘WD domain, G-beta repeat’ (PF00400, 151 genes), ‘RNA recognition motif’ (PF00076, 117 genes), ‘7 transmembrane receptor’ (PF00001, 117 genes), ‘Cytochrome P450′ (PF00067, 99 genes), ‘Major Facilitator Superfamily’ (PF07690, 94 genes), ‘Ankyrin repeats’ (PF12796, 92 genes) and ‘Homeodomain’ (PF00046, genes) in the top 10 (Figure 3B and Table S5). Among these, trypsins are essential serine proteases for protein digestion, and cytochrome P450 enzymes are one of the largest gene families in all organisms and are well known to be involved in a variety of important functions, such as insecticide resistance and metabolic detoxification. And carboxylesterases (PF00135, 72 genes) are one of the major enzyme families involved in insecticide resistance (Table S5).

We also explored the KOG annotation for putative digestion-related genes, and revealed 320 genes for ‘E: Amino acid transport and metabolism’, 143 genes for ‘F: Nucleotide transport and metabolism’, 499 genes for ‘G: Carbohydrate transport and metabolism’ and 463 genes for ‘I: Lipid transport and metabolism’ (Figure S1).

### 2.3. The Distribution and Enrichment Function of Paralogous Genes

The genes of *M. usitatus* are relatively well distributed across the chromosomes, except for the repeat-rich regions that contain a low abundance of genes (Figure 2B). Strikingly, the repeat sequences were significantly enriched at one end of the chromosomes (either the start or the end of chromosomes) and showed extremely low abundance in the other regions. Surprisingly, the majority of the paralogous genes (3469 out of 4613) were located in the repeat-rich regions, and the distribution of paralogous genes and repeat sequences showed a significant positive correlation (R = 0.723), suggesting the potentially faster evolution of paralogous genes driven by TE (transposable element) activities (Figure 2B,C).

**Figure 4:**
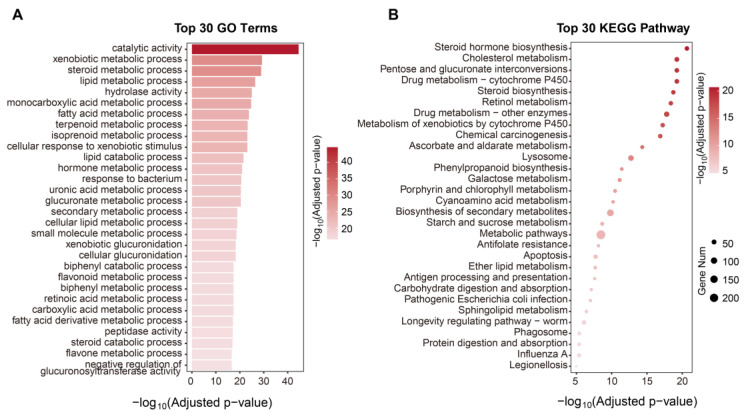
GO (**A**) and KEGG (**B**) enrichment of the paralogous genes in *M. usitatus*.

Furthermore, the functional enrichment analysis using GO and KEGG annotation for these paralogs revealed a strong association with several important biological functions, such as ‘hydrolase activity’, ‘peptidase activity’, ‘protein digestion and absorption’ and ’carbohydrate digestion and absorption’ for food digestion; ‘cytochrome P450′ and ‘response to bacterium’ for detoxification and resistance; and ‘Steroid hormone biosynthesis’ and ‘Longevity regulating pathway’ for reproduction (Figure 4A,B).

### 2.4. Comparative Genomics between M. usitatus and T. palmi

**Figure 5:**
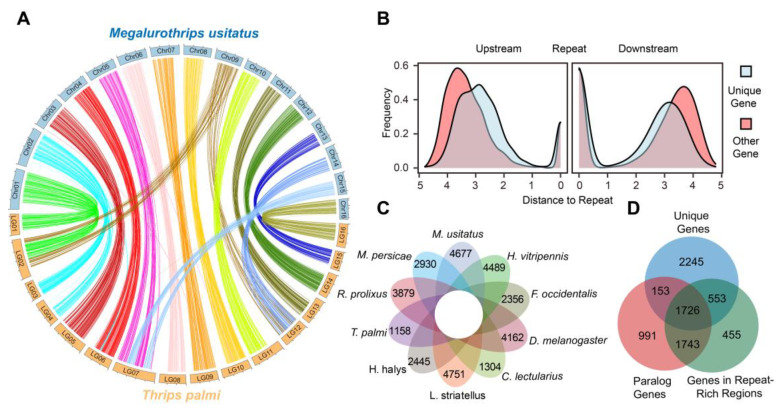
(**A**) The circos displays the synteny between *M. usitatus* and *T. palmi*. (**B**) The upstream and downstream distances of the unique genes and non-unique genes to the closest repeat elements were compared. X-axis represents Log10 (distance to repeat). (**C**) The flower chart displays the number of species-specific genes relative to the nine other species. (**D**) Venn plot shows the overlap among the unique genes, paralogous gene and genes in repeat-rich regions.

To further characterize the *M. usitatus* reference genome, we performed a comparative genomics analysis with *T. palmi*, the only Thripinae species with a published chromosome-level assembly [11]. Although *M. usitatus* and *T. palmi* are evolutionarily close, we identified only 973 synteny blocks containing 6367 orthologous gene pairs (accounting for only 34.2% and 40.8% of the total protein-coding genes, respectively) (Figure 5A), suggesting that the two Thripinae species have undergone extensive structural variations since divergence. In addition, their chromosomes show several large-scale recombination. For example, linkage group 7 (LG7) of *T. palmi* is a combination of Chr5 and Chr15 of *M. usitatus*.

Interestingly, we found that parts of the chromosomes (either the start or the end of chromosomes) have no collinearity between the two species, and these regions consistently match the repeat-rich regions in *M. usitatus*, indicating that these regions may be enriched with various mutations possibly driven by independent adaptive evolution. Furthermore, we found that the majority of the *M. usitatus* species-specific genes (4736 out of 7737) relative to *T. palmi* are located in the repeat-rich regions. The *M. usitatus* species-specific genes are preferentially close to repeat elements (Figure 5B). To confirm this result, we selected an additional eight close species and screened a total of 4667 *M. usitatus* species-specific genes (Figure 5C). More than a half of the genes in the repeat-rich regions are *M. usitatus*-specific genes (2279 out of 4477), and more than 75% of them (1726 out of 2279) are paralogs (Figure 5D), further indicating the close correlation between species-specific genes, paralogous genes and repeat elements in *M. usitatus*. Taken together, we hypothesized that the activities of repeat elements might be the major driving forces leading to the formation, expansion and mutation of *M. usitatus* species-specific paralogs.

### 2.5. Phylogenetic Analysis for M. usitatus and Evolutionarily Close Insecta Species

**Figure 6:**
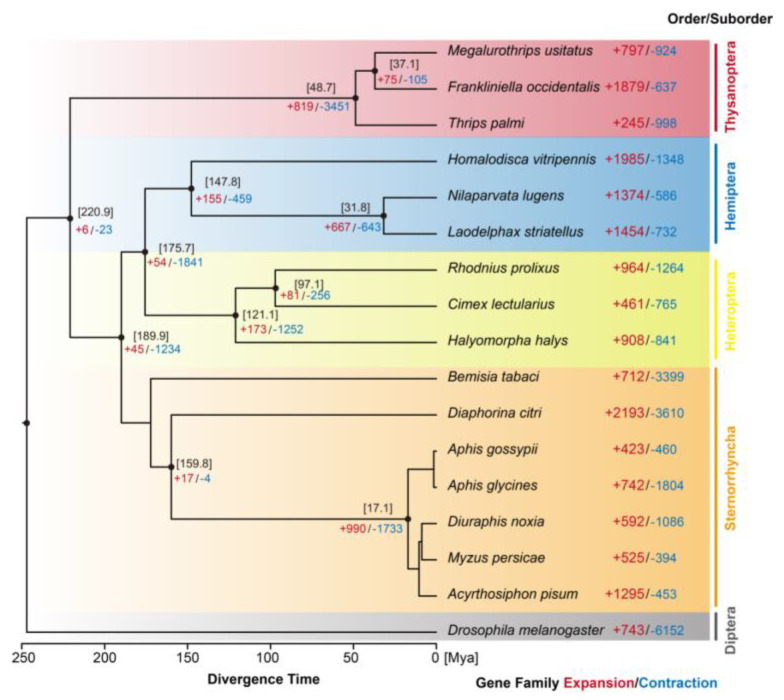
Phylogenetic tree of 17 Insecta species. Five distinct groups were classified based on order or suborder. X-axis represents the estimated divergence time (Mya). Numbers in square brackets represent the estimated divergence time (Mya). The red and blue numbers represent gene family expansion and contraction, respectively.

To investigate the phylogeny of *M. usitatus*, we selected 16 other insecta species and performed phylogenetic analysis with *Drosophila melanogaster* as an outgroup. In total, 93.6% of the total gene set derived from 17 Insecta species (277,742 out of 296,733) was classified into 28,066 orthologous groups. Among them, 513 orthologous groups containing single-copy genes were identified, which were further used to estimate the phylogenetic relationship. Finally, we constructed a phylogenetic tree with five distinct groups based on the order or suborder (Figure 6). Unexpectedly, the closest species to *M. usitatus* is *F. occidentalis*, with an estimated divergence time of 37.1 million years ago (Mya), followed by *T. palmi* with an approximate divergence time of 48.7 Mya.

### 2.6. Identification of Digestion-Related Genes

Digestion-related genes that encode the hydrolysis of proteins, lipids, carbohydrates and nucleic acids are essential for insects’ survival and development. Using both de novo Pfam-based and homologous-protein-based methods (122 digestion-related proteins reported in the tobacco hornworm, *M. sexta*, as seeds) [29], we identified 506 digestion-related enzymes including 422, 49, 32 and 3 hydrolases for proteins, lipids, carbohydrates and nucleic acids, respectively (Table S6), in *M. usitatus*. The 422 proteases can be further subdivided into 183 SPs, 12 cysteine proteases, 50 metalloproteases, 136 peptidases and 41 serine protease inhibitors (SPIs). Compared with genes in *M. sexta*, we identified 323 novel digestion-related genes, all of which are proteases, including 121 SPs, 6 cysteine proteases, 44 metalloproteases, 111 peptidases and 41 SPIs in *M. usitatus* (Table S6). In addition, proteases (162/422, *p* = 2.87 × 10^−10^), especially the largest subgroup serine proteases (96/183, *p* = 4.44 × 10^−16^) in *M. usitatus*, are significantly enriched in paralogs (Table S7). There is no statistical significance in the enrichment analysis of digestion-related genes of *M. usitatus* for TE association or unique gene (Table S7).

## 3. Discussion

In this study, we combined ONT and HiC technologies to generate a high-quality chromosome-level reference genome assembly (247.82 Mb) of bean flower thrips, *M. usitatus*, which is becoming the most destructive pest of legumes, such as cowpea *V. unguiculata* ssp. *sesquipedalis*, in southern China. The genome assembly consists of 16 chromosomes and 1 scaffold of unassembled contigs. We identified 15.05% repeats and 18,624 protein-coding genes, of which 4613 are paralogous genes and most are strongly TE-associated. Gene functional annotation revealed a set of digestion-related genes involved in the top terms of Pfam, GO and KOG, as well as significant terms of GO and KEGG in the paralog enrichment analysis. Integrating homologous and de novo strategies, we identified 506 putative digestion-related enzymes, the majority of which, 422, are proteases. Proteases and the largest subgroup serine proteases were statistically significantly enriched in paralogous genes. These results suggested that the diversity of paralogs driven by mobile elements may promote the expansion of digestion-related genes, especially SPs, in the evolution of *M. usitatus* adaptation to host plants, such as cowpea, with high protein content and abundant PIs. Our study will provide a valuable genomic resource for understanding the genetic variation among different pest insect species and their adaptation mechanisms to different host plants, and also provides a model case for gene family analysis.

A high-quality genome assembly is a key and fundamental requirement for biological research. Rapid development of sequencing technologies such as Illunima, ONT and Pac-Bio, assisting with scaffolding technologies such as HiC and Bionano, have increased continuity and accuracy of genome assembly. The first gap-free and T2T human genome assembly was completed in 2022, more than 20 years after the first draft version was published in 2000 [30]. Compared with previously reported genome assemblies of thrips, e.g., *F. occidentalis* [10] and *T. palmi* [11], our chromosome-level genome assembly has greatly improved continuity, the number of contigs decreased by 99% (187 vs. 74,788) and 85% (187 vs. 1324), and the length of contig N50 increased ~1800 (11.32 Mb vs. 6.04 kb) and ~20 times (11.32 Mb vs. 567 kb), respectively (Table S8). Our genome assembly still contains many gaps that need to be further addressed by PacBio HiFi sequencing technology to generate a gapless and complete T2T genome assembly in the future.

The diversity of insect digestive enzymes reflects the ability to digest and absorb nutrients, such as proteins, carbohydrates and lipids, etc., from the host plant, and the diversity of insect digestive enzymes is a critical factor affecting insect growth, development and reproduction [31,32]. Thrips feeding on a protein-rich host plant, such as cowpea, shortened their developmental period, expanded their lifespan and increased their egg production [33]. Compared to 122 digestive enzymes (85 proteolytic, 20 lipolytic, 16 carboxylolytic and 1 nucleolytic) identified in the tobacco hornworm (*M. sexta*) genome [29], the *M. usitatus* genome contains almost four times as many proteolytic enzymes (422, including 183 SPs and 136 peptidolytic enzymes) (Table S6). On the other hand, host plant legumes, such as cowpea, produce many protease inhibitors (PIs) that are negative against *M. usitatus*. The expansion of proteases, especially SPs, may reflect the adaptation mechanisms of bean flower thrips, *M. usitatus*, to the host plant cowpea as well as other legumes, i.e., overexpression of PI-sensitive proteinases, evolution of novel PI-insensitive proteinases or production of PI-hydrolyzing proteinases. The actual mechanism as well as the expression pattern of SPs during food digestion deserves further investigation.

Interestingly, besides abundant SPs, we also identified 41 SPIs (also called serpins) in *M. usitatus*, which is much more than the 34 serpins in the silkworm *Bombyx mori* [34], 32 in *M. sexta* [35], 29 in *D. melanogaster* [36], 25 in *Plutella xylostellam* [37], 18 in *Anopheles gambiae* [38] and 7 in *Apis mellifera* [39]. Serpins are effective inhibitors of insect SPs, adjusting the function of protein digestion enzymes when they are no longer needed and ensuring their harmony [40]. In addition, serpins perform various other physiological functions in insects like development, host–pathogen interactions and innate immune response.

## 4. Materials and Methods

### 4.1. Sampling

Adults of *M. usitatus* were obtained from cowpea, *V. unguiculata* ssp. *sesquipedalis*, in Lishui (28.45° N, 119.91° E), Zhejiang, China, in 2019. The population was reared on fresh cowpea in a climate-controlled chamber (25 ± 1 °C, 16 L: 8 days). About 200 adult thrips of mixed ages were randomly collected from the cultures. Briefly, adult thrips were rigorously decontaminated by immersion in 1% sodium hypochlorite solution (Gaide chemical, Hangzhou, Zhejiang, China) for 5 min, followed by rinsing in sterile water and immersion in 70% ethanol, and rinsing in sterile water. Before genomic DNA and RNA extraction, samples were transferred to liquid nitrogen and then stored at −80 °C.

### 4.2. Genome Sequencing and RNA-Seq

For genomic sequencing and RNA-seq, high-quality genomic DNA and mRNA were extracted and purified using QIAGEN DNA/RNA tissues kit (QIAGEN 69506/73404, Hilden, Germany), and prepared for sequencing libraries according to the manufacturer’s instructions for sequencing technology (Nextomics Biosciences Co., Ltd., Wuhan, China). ONT long-read sequencing was performed on the Oxford Nanopore PromethION platform, while NGS, HiC genome sequencing and RNA-seq were performed on the Illumina NovaSeq 6000 platform (Table S1).

### 4.3. Genome Assembly and Quality Assessment

The clean ONT long reads (GSA: CRX645666) were exported to NextDenovo (v2.4.0) [41] for de novo assembly with a range of expected genome sizes ranging from 250 to 300 Mb. The draft assembly with the longest contig N50 was selected for two rounds of base error correction performed by NextPolish (v1.4.0) [42], first using ONT long reads and then using NGS short reads (GSA: CRX645667). To scaffold the contigs to chromosome level, the clean HiC reads (GSA: CRX645668) were mapped to the polished contig assembly using bowtie2 [43], then 3D-DNA (v180114) [44] was used for scaffolding according to the HiC signal and finally Juicebox (v2.20.00) [45] was used to manually correct the chromosome based on visualization of the Hi-C contact map.

BUSCO (v5.4.4) [46] was used to assess the completeness of genome assembly using insecta_odb10 (https://busco-data.ezlab.org/v5/data/lineages/insecta_odb10.2020-09-10.tar.gz, accessed on 15 March 2023, n = 1367). In addition, the NGS short reads were aligned to the final genome assembly using bwa-mem2 [47] to evaluate genome completeness.

### 4.4. Repeat Annotation and Calculation of LAI

To obtain the repeat library of the *M. usitatus* genome assembly, Repeatmodeler (v2.0.3) [48] was used for de novo identification of repeat elements. The repeat consensus was then used as seed for RepeatMasker (v4.1.0) [49] to scan all associated repeat regions across the genome assembly. LTR_finder (v1.07) [50], LTRharvest (v1.6.2) [51] and LTR_retriever (v2.9.0) [52] were used to predict LTR retrotransposons. LAI, a reference-free genome metric for assessing genome assembly continuity, was calculated by LTR-retriever based on the intact LTR retrotransposons.

### 4.5. Gene Prediction and Functional Annotation

For gene structure annotation, we combined de novo (Augustus [53], Snap [54], Genemark [55], Glimmer [56]), homology-based (Exonerate (v2.2.0) [57], insecta_odb10), and RNA-seq-based methods (Trinity (v2.11.0) [58], RNA-seq data from pooled male and female adult thrips, GSA: CRX645669). Finally, Maker (v2.31.11) [59] was used to integrate the prediction results. The final gene set was functionally annotated using eggNOG-mapper (v2.1.9) [60] against databases including KOG (Eukaryotic Orthologous Groups, https://www.ncbi.nlm.nih.gov/research/cog, accessed on 3 April 2023), KEGG (Kyoto Encyclopedia of Genes and Genomes, https://www.genome.jp/kegg, accessed on 10 April 2023), CAZys (Carbohydrate-Active Enzymes, https://www.cazy.org, accessed on 15 April 2023) and Interproscan v5 [61] against Pfam (https://www.ebi.ac.uk/interpro/, accessed on 18 April 2023) and GO (Gene Ontology, http://geneontology.org/, accessed on 20 April 2023) databases.

### 4.6. Comparative Genomic Analysis

Jcvi [62] was used to identify the synteny blocks between *M. usitatus* and *T. palmi*. Orthofinder (v2.5.4) [63] was used to identify the orthologs among 17 Insecta species (Table S2), and paralogs within species and species-specific genes. Core single-copy genes present in all of 17 insecta species were obtained from Orthofinder results for further phylogenetic analysis. The Protein sequences of core single-copy genes were multiple-sequence-aligned by Mafft (v7.310) [64], then the conserved blocks of the multiple sequence alignments were identified by Gblock (0.91b) [65] and finally the phylogenetic tree was constructed by Raxml (raxmlHPC-PTHREADS-SSE3, v8.2.12) [66] and visualized with Figtree (v1.4.3, https://github.com/rambaut/figtree, accessed on 25 April 2023). The divergence time was estimated by Timetree5 [67] and mcmctree (v4.9) [68]. Expansion and contraction of gene family was analyzed using cafe (v4.2.1) [69].

### 4.7. Digestion-Related Genes

Sequences of all digestion-related proteins that hydrolyze proteins, lipids, carbohydrates and nucleic acids, reported in the tobacco hornworm *M. sexta* [29], were collected as seeds. Hmmer (v3.3.2, http://hmmer.org/, accessed on 30 April 2023) was used for searching putative digestive proteins against whole-genome proteins of *M. usitatus* (identity > 30%, length > 50, E-value < 10^−6^). Since protein hydrolases are the major component of digestion-related proteins, we tried to identify as many proteolytic enzymes as possible using Pfam domains of known digestion-related proteins from *M. sexta* reanalyzed by Interproscan v5 [61].

## 5. Conclusions

Bean flower thrips (*M. usitatus*) is one of the most destructive pests of legumes, such as cowpea *V. unguiculata* ssp. *Sesquipedalis*, in southern China. In this study, we reported a chromosome-level genome assembly and a functional gene annotation of *M. usitatus*, systematically identified a set of digestion-related genes and found a boom of proteases, especially SPs and SPIs, which may reflect the evolutionary mechanism by which *M. usitatus* adapts to feeding on high-protein host plants also with high SPIs. The reference genome of *M. usitatus* presented here will be a valuable genetic resource for understanding the key biology of the pest, such as host plant adaptation, genetic variation, resistance to pesticides and immunity to microorganisms such as viruses, bacteria and fungi, which will help to develop more effective and environmentally friendly pest prevention and control measures against *M. usitatus*.

## Figures and Tables

**Table 1:** Genomic statistics of *M. usitatus*.

Genome Features	Values
Total Size of Contig Assembly (bp)	262,628,421
Contigs	187
Contig N50 (bp)	11,321,337
Contig N90 (bp)	509,537
Total Size of Scaffold Assembly (bp)	247,822,653
Scaffolds	17
Maximum Length of Scaffold (bp)	21,644,367
Scaffold N50 (bp)	14,859,349
Scaffold N90 (bp)	11,714,690
GC Content	55.40%
Genes	18,624
Average Gene Length (bp)	2908
Average Exon Number Per Gene	5.23
tRNAs	3673
Repeat Content	15.05%
LTR Assembly Index (LAI)	11.61
BUSCO genome completeness	98.60%

## Data Availability

Genome data sets generated in this study were deposited in China National Center for Bioinformation (CNCB) (https://ngdc.cncb.ac.cn/) under Bioproject PRJCA015931. The chromosome-level genome assembly and gene annotations of *M. usitatus* have been stored in the GWH (Genome Warehouse, https://ngdc.cncb.ac.cn/gwh) database under accession GWHCAYF00000000. The raw sequence data sets, including ONT and NGS genomic reads, Hic reads and RNA-seq reads, were deposited in the GSA (Genome Sequence Archive, https://ngdc.cncb.ac.cn/gsa) database under accession CRA010459.

## Supplementary Materials

The supporting information can be downloaded at https://www.mdpi.com/article/10.3390/ijms241411268/s1.
