# Supplementary figures and images for: The Chromosome-Level Genome Assembly of Bean Blossom Thrips (Megalurothrips usitatus) Reveals an Expansion of Protein Digestion-Related Genes in Adaption to High-Protein Host Plants
Source: Int J Mol Sci. 2023 Jul 10;24(14):11268. doi: 10.3390/ijms241411268 (PMC10379191; doi:10.3390/ijms241411268)

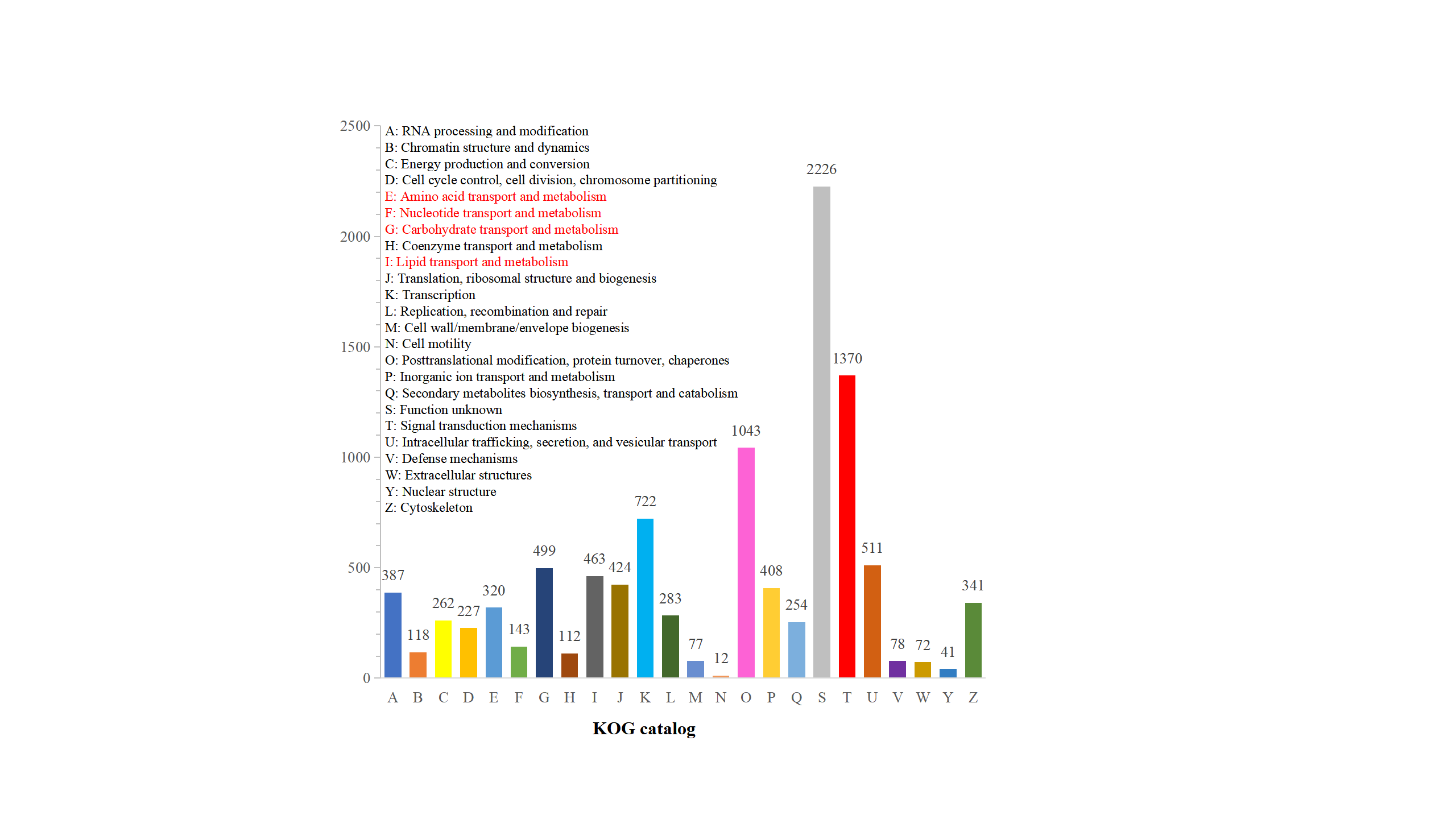

Supplement: Supplementary file 1 [file ijms-24-11268-s001.zip › Figure S1.tif]
